# The Development and Evaluation of the Nursing Leadership Excellence in Practice Program (L-EPP)

**DOI:** 10.3390/healthcare13111298

**Published:** 2025-05-29

**Authors:** Mitchell Dwyer, Kylie Chilcott, Samantha Finn, Kylie Sih, Jennifer Codee, Andrea Middleton, Pieter Jan Van Dam

**Affiliations:** 1 Tasmanian School of Medicine, UTAS Health, University of Tasmania, Medical Sciences Precinct, 17 Liverpool St., Hobart, TAS 7000, Australia; 2 Centre for Education and Research (Nursing & Midwifery), Tasmanian Health Service—Hospitals South, 25 Argyle Street, Hobart, TAS 7000, Australia; kylie.chilcott@ths.tas.gov.au (K.C.); samantha.sargent@ths.tas.gov.au (S.F.); kylie.sih@ths.tas.gov.au (K.S.); jennifer.codee@ths.tas.gov.au (J.C.); andrea.middleton@ths.tas.gov.au (A.M.); 3 School of Nursing, UTAS Health, University of Tasmania, Medical Sciences Precinct, 17 Liverpool St., Hobart, TAS 7000, Australia; pieter.vandam@utas.edu.au

**Keywords:** leadership, attrition, professional development, succession planning, capacity building

## Abstract

**Background:** Nursing leadership is associated with a host of benefits for patient outcomes and health services. Pressures relating to the COVID-19 pandemic saw many relatively inexperienced nurses thrust into leadership roles, often with little notice. In response to this situation, The Tasmanian Health Service—Hospitals South created the Leadership Excellence in Practice Program (L-EPP) as a way of developing the leadership skills of its nurses and midwives. This study aimed to describe the development of the L-EPP and to evaluate it from the perspective of its participants and their peers. **Methods**: A longitudinal mixed-methods study was conducted using data from the first two offerings of the L-EPP. The L-EPP employs a blended learning model comprised of e-learning, work-integrated learning and face-to-face workshops. Surveys targeting the participants’ leadership abilities were completed at numerous time points by participants themselves, their peers, and their managers. **Results**: A total of 57 participants completed the program. Workshop surveys indicated that these sessions were generally well-received by participants. Significant improvements were observed in several domains of leadership, from the perspective of the participants themselves and their peers and managers. **Conclusions**: The program was well-received by its participants, and would serve as a useful template for other organisations seeking to build the leadership capacity of their nurses and midwives. This may be particularly useful to organisations seeking to upskill their existing staff and prevent further attrition of nurses and midwives in the wake of the pandemic.

## 1. Introduction

At a broad level, leadership can be defined as “a process whereby an individual influences a group of individuals to achieve a common goal” [1] (p. 3). Leadership is a contextual phenomenon, meaning that factors such as the current organisational climate and team dynamics will influence which styles of leadership are most effective [2]. In a nursing context, effective leadership has long been associated with a host of positive outcomes, including improved patient outcomes [3,4,5], job satisfaction [6] and organisational performance [7]. The need to promote nursing leadership development has been recognised by several professional bodies (e.g., the International Council of Nurses, and the Australian College of Nursing) who now offer leadership development programs to their members [8,9]. Such courses often contain a didactic component as well as ongoing support to participants (e.g., in the form of mentoring, and career coaching) [8]. While such programs are undoubtedly beneficial, there is a recognition that leadership as a context-specific phenomenon, is something that must also be developed and delivered in-house (e.g., by supplementing learnings with workshops held on-site) [10]. In-house programs also have the advantage of being tailored towards the organisation’s needs and are delivered by people who are familiar with current challenges faced by the organisation [11].

The arrival of the COVID-19 pandemic in 2020 presented another set of challenges to the development of leadership in the profession of nursing. Faced with higher rates of illness and staff burnout, many nurses left the industry [12], in what some commentators have referred to as ‘The Great Attrition’ [13]. One observed effect of this attrition was that many relatively junior nurses were required to take on roles vacated by their more senior counterparts, often at short notice [14]. In Tasmania, Australia, it was recognised that nurses and midwives in this category who were employed by the state’s health service could benefit from leadership development opportunities, given that prior to the pandemic, such opportunities were primarily targeted towards more experienced nurses and midwives. This observation was supported by a 2020 systematic review of leadership development programs delivered in Australian hospitals [15] which found that most programs aimed to upskill nurses who were already in leadership or managerial roles, as opposed to fostering new leaders. The same review highlighted that little emphasis had traditionally been placed on the formal evaluation of programs, with most studies limiting their focus to the satisfaction of program participants [15]. This is problematic, given the large investment required to develop and conduct such programs [16].

In light of this perceived gap in the literature and the emerging challenges noted above, the Tasmanian Health Service—Hospitals South (THS-S) developed its Leadership Excellence in Practice Program (L-EPP) as a tailored solution for its local setting. The aim of the current study is to describe the development of the L-EPP and to evaluate the L-EPP from the perspective of the initial cohort of participants who completed the Program and their peers. Conducting this study will help to refine the L-EPP itself, while also aiding other health services seeking to develop similar programs.

### Background

The L-EPP was created by a team of Clinical Nurse Educators (CNEs) from the Tasmanian Health Service—Hospitals South’s (THS-S) Centre for Education and Research- Nursing & Midwifery (CERNM) throughout the course of 2021. This team of CNEs (herein referred to as the development team) also co-authored this manuscript. The L-EPP is underpinned by Transformational leadership, as the Tasmanian Health Service has committed to seek ‘Magnet’ accreditation. Magnet is a North American nursing program that strives towards an innovative work culture that leads nursing and midwifery in the provision of high-quality care [17]. One of the components of Magnet is transformational leadership, regarded as one of the most effective leadership styles in health services. In Transformational leadership, a leader mobilises employees’ motivations toward a shared vision by inspiring them to perform at a high level to produce better patient and organisational outcomes [18]. The L-EPP was created using an existing framework developed by the CERNM in 2019, known as the Excellence in Practice Program (EPP) framework. The EPP framework itself was created following an internal review of the CERNM’s activities, which considered how the unit could improve major clinical and professional issues in the health service. The review identified that many of the educational programs being delivered were not being thoroughly evaluated afterwards, and hence, there was a need for a new framework with a focus on evaluation.

Under the EPP framework, focused learning experiences are provided over a 3-month period, using a structured model of blended learning, work-integrated projects (WIPs), and supported and self-directed learning [19]. A blended learning model was chosen for several reasons. Providing foundational and theoretical learning through online delivery means that the valuable face-to-face time in the workshops can be focused on interactive activities and group-based learning. The online learning components can be self-paced between workshops, which can address some of the barriers nurses and midwives may have in accessing continuing education, including workload demands, shift work, and staff shortages [20]. The content of the L-EPP was determined following an extensive literature review of current best practices, and also consultation with stakeholders within the THS-S (e.g., in-house subject matter experts, potential participants).

A WIP was incorporated into the framework to provide an opportunity for translating the knowledge and skills acquired into practice. According to Frenk et al., transformative educational models that integrate theoretical learning with practical application are essential for preparing health professionals to meet the complex demands of modern healthcare systems [21]. The authors emphasise that such approaches foster continuous professional development and lifelong learning, which are essential for maintaining high standards of patient care and adapting to evolving healthcare environments [21]. By engaging in WIPs, nurses and midwives can directly apply their learning to real-world clinical settings, thereby enhancing their competencies and improving health outcomes. This hands-on experience not only solidifies their theoretical knowledge, but also equips them with the skills necessary to navigate the practical challenges they will face in their professional roles [21].

## 2. Materials and Methods

### 2.1. Design

A longitudinal mixed-methods study was conducted using survey data relating to two consecutive offerings of the L-EPP; the inaugural Program in 2021 and its second iteration in 2022. Quantitative and qualitative survey data were analysed separately and then presented using Kirkpatrick’s Four Levels of Training Evaluation model [22]. A longitudinal design was chosen to measure changes in learners’ behaviour (a key aspect of Kirkpatrick’s model) [22]. Behaviour changes in the context of this study are defined as the degree to which participants apply what they learned during the L-EPP when in practice [23]. Furthermore, Kirkpatrick’s model provides a clear structure for how outcomes can be measured; allowing organisations to improve their educational interventions [24]. Appendix A shows the flow of participants through the L-EPP in both years.

### 2.2. Setting

The THS-S includes the Royal Hobart Hospital, which is the largest of four tertiary care centres in the Australian state of Tasmania, with a capacity of 566 beds [25]. As of 2019, there were 1662 nurses and 106 midwives employed by the THS-S.

### 2.3. Sample

The study used a purposive sample, whereby all nurses and midwives employed by the THS-S at Grade 2–4 level (including those currently working on a casual/per-diem basis) were sent an email inviting them to submit an expression of interest (EOI) in completing the L-EPP. This cohort was targeted following a needs assessment of existing leadership development opportunities within the organisation, which highlighted that no opportunities were currently available to this group. Nurses and midwives employed by external agencies were not targeted, as it would be difficult for such individuals to complete WIPs mandated by the program. Nurses and midwives were selected for this program as it is the remit of the development team to provide educational opportunities to this group. Details of the L-EPP were also advertised on the THS-S intranet. This intranet page and the invitation email advised recipients that they were eligible to participate in the L-EPP if they were a direct care enrolled or registered nurse or midwife employed at Grade 2–4 level with greater than 12 months’ experience, following the initial date of registration in their profession. Participants needed the endorsement of their line manager in order to apply, and also needed to agree to pay $120AUD to complete a suite of online learning modules delivered by the Australian College of Nursing (ACN), which are discussed further below. An optional strengths assessment and coaching session were also offered to participants in this email, at an additional cost of $30AUD.

In addition to the above criteria, applicants were required to answer a series of questions about what they hoped to gain by undertaking the L-EPP (i.e., their learning objectives). Applications were then assessed on the perceived benefit each applicant would obtain from completing the L-EPP. Applicants whose learning objectives could not be met by the L-EPP were referred to more suitable learning opportunities available in the organisation. Consideration was also given to the participants’ roles and workplaces, to ensure that the cohort was diverse and broadly representative of the wider population of nurses and midwives in the THS-S. A total of 57 participants commenced the L-EPP during the study period (30 in 2021, 27 in 2022) who were the focus of this evaluation study. Enrolments were capped at this number in both years, as this was deemed to be the maximum number possible whilst still providing participants with an optimal experience.

### 2.4. The Leadership Excellence in Practice Program

The L-EPP employs a blended learning model comprised of three components: (1) e-learning, (2) work-integrated learning (WIL), and (3) face-to-face workshops, all of which were facilitated by the development team. As noted above, the e-learning component of the L-EPP was comprised of three online modules hosted by the ACN. These modules, which are listed below, were chosen as they were readily available and evidence-based resources which the team deemed suitable for the THS-S’s organisational context. It was also hoped that by becoming members of the ACN and accessing its library of resources, participants would be more likely to undertake modules on other topics and engage with this professional body more broadly. The timing of these modules was designed to align with the content of each upcoming workshop. Accordingly, participants completed the first module before the first workshop, the second module before the second workshop, and the third before the third workshop.

- Module One: Foundational Concepts in Nursing Leadership
- Module Two: Staff Engagement and Communication
- Module Three: Professional Issues in Contemporary Nursing

The WIL phase required L-EPP participants to develop and undertake a relevant WIP within their workplace, which was based upon an identified need in their workplace. Three face-to-face workshops were held 4–6 weeks apart. Participants were issued with a set of pre-reading documents to prepare themselves for each workshop. Workshops one and two each consisted of seven interactive sessions on various topics delivered by the development team and a number of experienced leaders from the broader Tasmanian healthcare system.

The third workshop was comprised of five-minute presentations from each of the participants about the process they took to conduct their WIL projects, followed by five minutes of question time from the audience. This day concluded with an address from experienced leaders from various levels of the healthcare system. The delivery of the 2021 workshops was slightly refined using participant feedback prior to the 2022 offering. Specifically, one session was altered after participants in the 2021 cohort indicated that its content was pitched at too high a level for them. Workshop topics and learning outcomes otherwise remained the same in both years.

Several activities also took place between workshops. Firstly, each member of the development team was allocated 5–6 participants to provide support during the Program. In addition to being a point of contact for any queries, team members also held a series of optional online ‘drop-in sessions’ for their allocated participants, where attendees could ask questions of the team member and their fellow participants (e.g., “how can I access data to inform my WIP?”). These sessions were also used as an opportunity to develop participants’ critical thinking skills. Further opportunities for participants to network with each other were made possible by using a ‘Learning Hub’ intranet page, where participants could post queries to, and collaborate with, other L-EPP participants. The hub also provided updates from facilitators, and access to presentations and other learning resources from the workshops.

Between workshops two and three, participants had the option to complete a ‘Clinical Moment’ reflective piece, in which they reflected on an episode of leadership in practice. Participants who underwent the strengths assessment mentioned above were offered a coaching session with a member of the development team (KS; an accredited coach), in which they could explore their identified strengths, and how these are expressed in their place of work and their leadership journey. Lastly, participants also took part in a compulsory ‘buddying’ activity, whereby each participant selected another participant to interview about their objectives in undertaking the Program, and the nature and challenges of their leadership journey. Appendix B displays the L-EPP’s 13 learning outcomes which were mapped to the Program’s three modes of delivery mentioned above. Participants were not required to pay any money in order to take part in the program, however, they did stand to gain from their participation through the acquisition of new skills.

### 2.5. Data Collection

Participants completed surveys addressing their own leadership capabilities at baseline (i.e., prior to their first workshop) and at follow-up, which occurred three months after the third workshop in both years. This interval was used to allow sufficient time for the participants’ learning to be embedded. The questions for this survey required participants to rate their perceived ability in 22 aspects of leadership, 16 of which were Zenger and Folkman’s leadership competencies [26] with the remainder being created by the development team. The rating of competencies was done using a 10-point scale (10 being the highest rating, 1 being the lowest). Surveys addressing the same leadership competencies, albeit from the perspective of the participant’s current manager and a peer-nominated by the participant, were also completed at baseline and follow-up.

Surveys in relation to each individual workshop were completed by participants during a 30-min window set aside at the conclusion of that workshop. These required participants to rate the presentation and time (duration) of each workshop session using 4 and 3-point Likert scales, respectively. The content of each workshop session was rated using binary responses of ‘relevant’ and ‘irrelevant’. Paper surveys were used in the 2021 Program, while the 2022 Program used a QR code linked to an online survey portal, which participants could scan and complete using their devices. Each workshop survey also contained five free-text questions, targeting which aspects of the day participants felt were most/least useful, and the extent to which they had learned skills which could be applied in their workplaces. An ‘overarching’ survey was distributed to participants upon completion of their third workshop, which addressed the program as a whole. These surveys consisted of 11 free-text questions, which targeted the overall impact the Program had on participants across a number of domains (e.g., skills acquired, behaviour changes). Each of the above surveys was created by the development team.

### 2.6. Data Analysis

All data were analysed upon completion of the second offering’s follow-up period by two academics. These academics were selected on account of their expertise, and because they were not involved in the development, delivery or ongoing administration of the L-EPP. Data relating to both the 2021 and 2022 iterations of the L-EPP were pooled together for analysis. Quantitative data were analysed in IBM SPSS Version 29.0 (Armonk, NY, USA) using descriptive statistics (frequencies, means, percentages). Paired samples *t*-tests were also used to determine whether there had been improvements in participants’ leadership abilities from baseline to follow-up. Missing pre or post-data (e.g., from participants, their peers or managers failing to return baseline and/or follow-up surveys) were excluded pairwise. Qualitative data were analysed using a thematic analysis framework [27]. The analysis involves the following stages: code material; identify themes construct thematic networks; describe and explore thematic networks; and interpret patterns. This inductive analysis is carried out by developing basic themes, organising themes, and overarching global themes, in combination with a deductive approach, whereby the first three levels of Kirkpatrick’s model [22] served as global themes: Reaction, Learning and Behaviour. This hybrid approach of using both inductive and deductive analysis complements each other, and prevents salient data from being missed, thus increasing the rigour of the analysis.

#### Kirkpatrick’s Evaluation Framework

Once all quantitative and qualitative data had been analysed, the data were merged and presented using Kirkpatrick’s evaluation framework, which assesses training and education programs using a four-level approach [22]. For the purpose of the current study, only the first three levels of Kirkpatrick’s model were utilised. Table 1 shows what is targeted by each of the first three levels of Kirkpatrick’s model, along with which data sources were used to inform each level in the current study.

## 3. Results

### 3.1. Description of Sample

The inaugural L-EPP ran from May to August 2021, while the second iteration ran slightly longer (from May until November 2022), owing to the need to postpone one workshop due to the effects of COVID-19. Of the participants who commenced the L-EPP in both years, 27 followed it through to completion in 2021, while only 17 completed the 2022 L-EPP. This attrition resulted in missing data for the pre-post appraisals. After missing data were excluded pairwise, the appraisal data from the participants themselves, their peers, and managers, had matched pairs of N = 27, N = 12 and N = 14, respectively. Most (>90%) participants were female and were registered nurses or midwives employed at the Grade 3–4 level, with only two participants who were Grade 2 level enrolled nurses. Participants in both years were employed in a variety of different areas within the health service, with representation from a host of medical, surgical, a community-based teams, along with mental health. In both years, twelve participants opted to undertake the abovementioned strengths assessment and coaching.

### 3.2. Kirkpatrick’s Model Level 1—Participants’ Reaction to the L-EPP

Table 2 summarises the participants’ reactions to the sessions comprising each of the L-EPP’s three workshops. The content of the workshops was almost unanimously (97%) regarded as being relevant. A majority of respondents (57%) stated that the presentation of the workshops was excellent, while the number of respondents who found the presentations to be inadequate was negligibly small (<1%). Most respondents (84%) found the duration of sessions within the workshops to be adequate, however, there were smaller numbers of participants who found the duration of some sessions to be inadequate or excessive (4% and 11% of respondents, respectively). Table 3 presents the qualitative data analysis including global themes, categories and codes. The global theme of value adding and waste aligned well with level 1 of Kirkpatrick’s model, as participants expressed their reaction to the L-EPP. Participants felt that the presenters and educators encouraged them to learn from others. This learning was identified when participants spoke about the health service improvement projects they carried out as part of the Program.

*Some of the other projects… are applicable to our unit; they have been inspiring and increased my awareness of other gaps within my unit.*

Other participants articulated that the Program added value to their professional life. This was expressed as becoming a mindful leader who could make a difference.

*It felt practical and relevant and made me feel like I could achieve something that would make a difference.*

One participant noted that some of the content related to Quality and Safety was duplicated from an existing in-house Quality and Safety Course.

*I have already covered most of the content in other workshops.*

Participants expressed that a small number of components of the Program were not value-adding and improvements were required. One such component was the session in relation to evidence-based practice and leadership.

*I feel I got lost in the presentation and the different abbreviations and systems.*

### 3.3. Kirkpatrick’s Model Level 2—Learning

The global theme ‘Learning to see’ entails skills that developed during the Program, which involves assessing the current state of care provision issues, and after an issue has been identified, a plan is developed to address the issue. This was followed by actions taken to implement improvements.

*This leadership course—helped me see improvements that could be made in our unit and now I can influence, motivate, and inspire others.*

The Program helped participants to understand themselves as leaders and it increased their awareness of what a leader should or should not be. It gave them insight into skills they would like to learn, and it changed their views about leadership.

*This Program changed my point of view about a leader. I got more clear understanding of leadership qualities.*

It appeared that collaboration was a central component of the Program, encouraged by the opportunities for participants to work together and through recognising each other’s experience and ways of working.

*Identifying all the different personalities and experience of others and importance to ask/seek multiple viewpoints to get good understanding.*

*This program has helped me step outside my comfort zone and engage with my peers, a skill I have been able to transfer to clinical practice.*

### 3.4. Kirkpatrick’s Model Level 3—Behaviour

Table 4 summarises the participants’ improvements in various leadership capabilities, from the perspective of the participants themselves, their nominated peer, and their current manager. Participants reported significant improvements in all but one domain ‘Is comfortable with, and values input of experts’. Ratings by the participants showed improvements in all 22 domains, however, only five of these were statistically significant. Ratings from participants’ managers showed a similar pattern; while some degree of improvement was observed in all 22 domains, only 14 of these results were statistically significant. There were no instances where participants (either from their own perspective, or that of their peers or managers) were found to have a decreased capability over time. The global theme ‘understanding leadership’ includes the codes being-self-aware, being reflective, seeking others and changing views. Particularly, self-awareness and seeking others played a large role in understanding leadership, as participants started to recognise personality traits within themselves and other people. This recognition of others became clear through actively seeking mentors.

*Thinking and seeking out important people. ‘Mentors’ for different aspects in working life will help me to have multiple viewpoints.*

Many participants learnt how to become better leaders, and they articulated this by having gained enhanced knowledge and skills, building on their existing leadership foundation.

*I have a foundation to build upon my understanding and practice in leadership.*

Completing the Program increased participants’ confidence, and many felt better equipped to help champion change in their workplace and to handle conflict. 

There are challenges within in my workplace that I feel prepared to manage now.

*I am more able to reach positive outcomes in conflict and scenarios which prior may have overwhelmed me.*

The final global theme ‘collaborative ways of working’ involves the motivation to work together and contributed to building networks and sharing of knowledge.

*I have been establishing connections with staff outside my practice area by sharing knowledge about things that I use in my area.*

## 4. Discussion

This study evaluated a leadership program developed for direct care nurses and midwives. Our findings demonstrate that this program, which was designed and delivered by in-house CNEs, has greatly benefited the initial cohort of participants who undertook it. These findings also indicate that the EPP framework is an appropriate platform for providing education on leadership development. In relation to Level 1 of Kirkpatrick’s Model (reaction), it is evident that the Program was generally very well-received by the participants. The main criticism in this regard came from a relatively small number of respondents who found the duration of certain workshop sessions to be excessive, with a smaller number reporting that the sessions were too brief. Some participants found the content of the session on evidence-based practice to be in need of improvement. On reflection, this session may have too been complex for nurses and midwives working in direct care roles, and perhaps would have been better pitched towards a more appropriate level.

Central to Level 2 of Kirkpatrick’s model (learning) [22] was the evidence of collaboration between participants, contributing to effective networks and leadership development. This is consistent with Paterson and colleagues, who found that nurses in their leadership program who shared thoughts and reflections, contributed to clarity and coherence in relation to continuous building of personal leadership capacity [28]. The Program’s WIPs contributed to creating insights and learning, and it is important to note that the benefit of these projects will likely be reaped by both the participants themselves (e.g., in the form of increased confidence and competency) and to the wider organisation, in the form of improved systems and processes consistent with other studies investigation the outcomes of WIPs [29,30].

The high number of significant improvements in leadership capabilities reported by the participants may be partly due to participants initially underestimating their own abilities. Indeed, the participants’ appraisal of their baseline ability to create a SMART goal was the lowest mean rating given by any party at any timepoint, and by a substantial margin. This tendency to downplay one’s abilities has been shown to be particularly pronounced among women [31], who comprised a majority of the current study’s participants. The lack of significant improvements in participants’ leadership capabilities from the perspective of their peers is likely due to the fact that, in many cases, baseline ratings were quite high. This is exemplified in the findings for ‘Is an asset, value adding to the team and organisation’ and ‘Practices self-development’, which, despite having mean ratings at follow-up of over 9/10, were both statistically non-significant improvements. This finding is in contrast with other studies where supervisors and peers reported on participants’ improvements in performance as a leader [32,33]. It is unknown why peers in this study ranked the leadership capabilities high and this warrants further investigation.

As Australia’s only island state, Tasmania was able to effectively insulate itself from the initial wave of COVID-19 cases by preventing all non-essential travel into the state. This policy was relaxed in December 2021, when the state government reopened borders to all arrivals from interstate and abroad, resulting in a sharp increase in the number of COVID-19 cases in the proceeding months. This increase, and the resulting strain on the state’s health service, occurred during the running and follow-up period of the 2022 L-EPP, and is a likely explanation for the high rates of missing data observed in the peer and manager surveys for that year. For instance, it is possible that participants were unwell themselves, or had otherwise disengaged from the Program after the final workshop day, and were reluctant to ask their peers or managers to complete a non-essential task such a survey during this busy period. This phenomenon was observed elsewhere during the COVID-19 pandemic, whereby healthcare facilities were asked to restrict all non-essential activities [34]. The post-COVID-19 era has been characterised by high rates of staff turnover, burnout, and a considerable number of nurses and midwives leaving the industry altogether [35]. The local context in which the L-EPP took place has not been immune to these forces, with rates of anxiety, depression, and post-traumatic stress disorder being reported previously [36]. The arrival of the L-EPP in the midst of the pandemic likely played a role in decreasing staff members’ intention to leave, as it represents a much-needed form of organisational support [37]. The L-EPP serves as a prime example of how other healthcare organisations can enhance their workforce capacity by implementing a similar program, as the design of the L-EPP is potentially a good fit for organisations who share a passion for leadership development and strive to retain well educated nursing staff.

This study has limitations which must be acknowledged. As noted above, data in relation to Level 4 of Kirkpatrick’s model (Results) are not presented in the current study. Given that the study participants represent only a small portion of the organisation’s workforce, it would be ill-advisable to draw links between the L-EPP and changes in broader system performance, such as reduced costs and improved quality. Further research is needed to look at the L-EPP’s impact on ‘Level 4a’ as described by Frich and colleagues [38], focusing on organisational results from the perspective of the participants and their subordinates (e.g., in terms of their job satisfaction, career progression and staff retention) among other factors. A second limitation relates to the fact that the study did not employ a control group, and hence, it is possible that reported improvements may be partly attributed to factors other than participation in the program. Another limitation of our study is that the surveys completed by participants at the close of each workshop had not been validated prior to being used.

## 5. Conclusions

Structured leadership development opportunities at the level of clinical nursing and midwifery are essential to improving patient and organisational outcomes. The development of the L-EPP and its longitudinal evaluation have demonstrated that participants have made improvements in almost all domains of leadership development. This, in turn, suggests that the Program has made a difference in building leadership capability within the THS-S, and potentially preventing some attrition of its staff. It is hoped that the success of this Program can be replicated in other health services within Tasmania and Australia, many of whom would be facing similar challenges.

## Figures and Tables

**Table 1:** Levels of Kirkpatrick’s Model and Accompanying Data Sources.

Level of Model	What is Measured	Applicable Data Sources
**Level 1—Reaction**	Participants’ initial perception/impressions of the Program.	Individual workshop evaluation surveys (3 per iteration)
**Level 2—Learning**	How the education has improved/developed knowledge, skills, professional attributes, or attitudes	Individual workshop evaluation surveys (3 per iteration) Overall L-EPP evaluation surveys (1 per iteration)
**Level 3—Behaviour**	How the education is being applied by the participant	Self-review baseline and follow-up surveys Peer review baseline and follow-up surveys Manager review baseline and follow-up surveys Overall L-EPP evaluation surveys (1 per iteration)

**Table 2:** Participants’ Reaction to the L-EPP.

	**2021 Program**	**Presentation**				**Content**		**Time**		
**Workshop**	**Session Title**	**Inadequate**	**Adequate**	**Good**	**Excellent**	**Irrelevant**	**Relevant**	**Inadequate**	**Adequate**	**Excessive**
W1 (n = 28)	Mindful Leadership		1	12	15	-	28	-	25	3
	What is Leadership?	-	-	7	21	-	28	-	28	-
	Leading Communication—Understanding How We Communicate	-	1	12	14	1	25	1	25	-
	Leading by Example Demonstrating Leadership in Practice	-	1	5	22	1	27	1	25	2
	Leading with Evidence Based Practice Continuous Improvement	-	9	14	5	1	27	-	25	3
	“What Gets Your Goat?” Setting SMART Goals	-	-	4	24	-	28	5	22	1
	Understanding Your Strengths	1	-	7	19	-	25	2	24	-
W2 (n = 25)	Quality and Safety	-	-	7	17	1	20	2	19	3
	ICN—Big Picture Thinking	-	2	12	10	1	23	-	21	3
	What Makes Us Tick?	-	4	10	9	1	24	2	18	4
	Culture—See/Feel/Hear (Iceberg)	-	-	6	18	-	24	-	22	2
	Values (Personal/Workplace/Organisation)	-	-	7	17	-	24	2	21	1
	Critical Thinking	-	-	4	19	-	24	-	22	2
	Qualities of Inspirational Leaders	-	-	6	-	-	24	1	21	2
W3 (n = 23)	Presentation—Chief Nurse	-	2	9	12	-	23	3	17	2
	Participant Presentations	-	-	5	17	-	22	-	19	3
	Afternoon tea—the benefits of culture, arts in healthcare	-	-	8	15	-	22	-	20	2
	Yearly Total	1	20	135	254	6	418	19	374	33
	**2022 Program**	**Presentation**				**Content**		**Time**		
	**Session Title**	**Inadequate**	**Adequate**	**Good**	**Excellent**	**Irrelevant**	**Relevant**	**Inadequate**	**Adequate**	**Excessive**
W1 (n = 26)	Mindful Leadership	1	-	10	15	-	26	-	22	4
	What is Leadership?	-	-	8	18	-	26	-	24	2
	Leading Communication—Understanding How We Communicate	-	1	10	15	-	26	-	25	1
	Leading by Example Demonstrating Leadership in Practice	-	1	15	10	-	26	-	23	3
	Leading with Evidence Based Practice Continuous Improvement	2	6	9	9	2	24	3	19	4
	“What Gets Your Goat?” Setting SMART Goals	-	3	13	10	-	26	-	22	4
	Understanding Your Strengths	-	2	8	16	-	26	-	22	4
W2 (n = 25)	Quality & Safety	-	2	13	10	1	24	4	17	4
	Project Thinktank	-	3	6	16	1	24	3	19	3
	Reflection—What Makes Us Tick? SCARF Model/DOPE TEST	-	-	12	13	-	25	2	19	4
	Culture—See/Feel/Hear (Iceberg)	1	3	8	13	-	25	-	18	7
	Values (Personal/Workplace/Organisation)	-	1	6	18	-	25	2	19	4
	Critical Companions	-	1	17	17	-	25	-	22	3
	Qualities of Inspirational Leaders	-	2	7	16	-	25	1	18	6
W3 (n = 13)	Meeting the Secretary and Head of Agency for the Dept of Health	-	1	6	6	2	11	1	9	3
	Participant Presentations	-	3	4	6	1	12	2	10	1
	Afternoon Tea Entertainment	-	-	6	7	4	9	-	12	1
	Yearly Total	4	29	158	215	11	385	18	320	58
		**Presentation**				**Content**		**Time**		
		**Inadequate**	**Adequate**	**Good**	**Excellent**	**Irrelevant**	**Relevant**	**Inadequate**	**Adequate**	**Excessive**
**Combined Total**		5	49	293	469	17	803	37	694	91
		0.61%	6%	35.91%	57.48%	2.07%	97.93%	4.5%	84.43%	11.07%

**Table 3:** Qualitative Data Analysis.

Global Themes	Categories	Codes	Supporting Quotes
**Value adding and waste**	Value adding (1)	Making a difference	It felt practical and relevant and made me feel like I could achieve something that would make a difference
		Learning about SMART Goals	
		Being a Mindful leader	Focusing my attention to certain things and releasing tension which may reduce my choices
		Bringing positivity and encouragement	I really appreciate how positive and encouraging all the presenters/educators were of us as individuals and the value we have/bring.
		Learning from others	We can all learn from each other and that I can improve for the better Listening to everyone’s informative projects. Great energy and harmony (3). Presentations of quality projects that can improve not just one department, but can be adopted to other departments/areas as well (3) Some of the other QI projects (i.e., dialyse, interpretative services) are applicable to our unit; they have been inspiring and increased my awareness of other gaps within my unit (3)
	Value adding (2)	Embracing Quality and Safety	Great to know what’s available and how
		Leadership Qualities	Qualities of Inspirational Leaders—it gave me ideas of qualities that I want to develop and some that I already have
	Room for Improvement (1)	(Evidence based practice) delivery	Whilst the presentation was valuable and well delivered, it was one of the slower presentations and had less impact than the others
			I feel I got lost in the presentation and the different abbreviations and systems
	Room for Improvement (2)	Duplicating materials	Quality and safety—mostly because I have already covered most of the content in another workshops
**Leadership to see**	Enhanced knowledge and skills (1)	Collective learning	Able to participate and learn, feel I am not alone, able to share
		Using my foundation	I have a foundation to build upon my understanding and practice in leadership
		Being reflective	This gives us more time to think about “leadership”, more in-depth to explore the quality of good leadership
	Enhanced knowledge and skills (2)	Embracing values	Linking personal vales with organisation values then linking culture and how they affect each other
	Enhanced knowledge and skills (3)	Receiving ideas	Hearing about other people’s QI projects has made me aware of what is possible and given me many ideas for future projects in my work area
	Applying my learning (1)	Focussing on collaboration	Today’s session would help me to actively engage all team members to achieve common goals and to build a sense of teamwork. Work on my characteristics/values to promote collaboration and inspiration in the workplace. This leadership course—helped me see improvements that could be made in our unit and now I can influence, motivate, and inspire others
		Increased confidence	I will be more equipped to help champion change in my workplace
	Applying my learning (2)	Feeling prepared	There are some challenges within my workplace that I feel prepared to manage.
		Being vulnerable	Having learnt more information about my personality type I can work on the weaker points.
	Applying my learning (3)	Being supportive	How to support peers regarding project ideas—and be a better mentor
**Understanding leadership**	Knowing self and others	Being self -aware	This program/workshop helped me to understand myself (as a leader) (that) who is not a traditional and not comply what a leader should or supposed to be. Insight into myself and others. Insight into things I want to learn about now
		Being reflective	
		Seeking others	Understanding the importance of networking. Thinking and seeking out important people. “Mentors” for different aspects in working life and personal life
		Changing views	This program changed my point of view about a leader. I got more clear understanding of leadership qualities. This program helped me grow in my leadership career and will help me to grow
	Reflective learning (O)	Thinking outside the box	
		Understanding strengths and self	In learning to recognise personality traits within myself and other people my ability to analyse situations has improved and I am more able to reach positive outcomes in conflict and scenarios which prior may have overwhelmed me
		Changed behaviours	In the context of analysing and critical thinking conversations and relationships with people at work. Analysing things like the SCARF model and tailoring my behaviour around it I have increased enthusiasm and spread it on my ward. More talk about clinical projects to guide our unit
	Driving change (O)	Encouraging	Identifying all the different personalities and experience of others and importance to ask/seek multiple viewpoints to get good understanding
		Being confident	
**Collaborative ways of working (0)**	Working together	Recognising others	Identifying all the different personalities and experience of others and importance to ask/seek multiple viewpoints to get good understanding
		Building networks	This program has helped me step outside my comfort zone and engage with my peers, a skill I have been able to transfer to clinical practice I have been establishing connections with staff outside my practice area by sharing with knowledge about things that I use in my area
	Motivating others (0)	Influence and inspire others	
		Focussing on collaboration	Today’s session would help me to actively engage all team members to achieve common goals and to build a sense of teamwork. Work on my characteristics/values to promote collaboration and inspiration in the workplace.

**Table 4:** Participants’ Pre-Post Improvements in Leadership Domains ^†^.

	Self Appraisal			Peer Appraisal			Manager Appraisal		
	Pre (N = 27)	Post (N = 27)		Pre (N = 12)	Post (N = 12)		Pre (N = 14)	Post (N = 14)	
Domain	Mean (SD)	Mean (SD)	*p*-Value	Mean (SD)	Mean (SD)	*p*-Value	Mean (SD)	Mean (SD)	*p*-Value
Takes Initiative	7.19 (1.24)	8.67 (1.00)	<0.001	7.75 (1.22)	9.08 (1.00)	0.013	7.86 (1.29)	8.36 (0.93)	0.169
Champions Change	6.26 (1.35)	8.41 (1.12)	<0.001	7.67 (1.88)	9.00 (1.21)	0.058	7.50 (1.51)	8.14 (1.29)	0.095
Drives for Results	6.59 (1.45)	8.44 (1.05)	<0.001	7.75 (1.60)	8.75 (1.36)	0.067	7.36 (1.22)	7.93 (1.39)	0.15
Practices self-development	7.59 (1.47)	9.00 (1.00)	0.001	8.67 (1.30)	9.25 (1.06)	0.206	7.86 (1.29)	8.50 (1.09)	0.045
Develops others	7.11 (1.40)	8.67 (0.88)	<0.001	8.08 (1.31)	8.50 (1.31)	0.318	7.14 (1.03)	7.93 (1.27)	0.035
Inspires and motivates others to high performance	6.89 (1.31)	8.38 (1.02)	<0.001	7.67 (1.92)	8.42 (1.51)	0.121	7.07 (1.39)	8.21 (1.25)	0.002
Builds relationships	7.70 (0.95)	8.74 (0.94)	0.001	8.33 (1.83)	8.92 (1.51)	0.171	7.50 (1.40)	8.43 (1.28)	0.026
Creates environment for team to achieve best work	7.70 (0.91)	8.52 (0.94)	0.002	8.08 (1.56)	8.58 (1.51)	0.191	7.71 (1.27)	8.21 (1.31)	0.131
Collaboration and teamwork	7.93 (0.92)	8.74 (0.94)	0.002	8.50 (1.62)	8.67 (1.56)	0.689	7.64 (1.15)	8.50 (1.29)	0.001
Connects the group to the outside world	6.37 (1.47)	8.00 (1.41)	<0.001	6.67 (2.02)	8.17 (1.90)	0.037	6.93 (1.39)	8.07 (1.59)	0.012
Trusted member, works for the good of the team	7.85 (1.10)	8.74 (1.23)	0.005	8.50 (1.31)	8.75 (1.66)	0.651	8.00 (1.36)	8.79 (1.19)	0.021
Adds value to the team and organisation	7.41 (0.75)	8.52 (1.28)	<0.001	8.92 (1.44)	9.08 (1.17)	0.689	8.36 (1.22)	8.79 (1.19)	0.212
Is comfortable with, and values input of experts	8.30 (1.66)	8.93 (1.69)	0.134	8.08 (1.78)	8.83 (0.94)	0.095	7.64 (1.39)	8.50 (1.35)	0.089
Solves Problems and Analyses Issues	7.22 (1.48)	8.52 (1.16)	<0.001	8.00 (1.60)	9.00 (1.21)	0.039	7.64 (1.34)	8.21 (1.42)	0.205
Resourceful, removes obstacles and solves problems	6.85 (1.70)	8.19 (1.18)	0.004	7.75 (1.71)	8.83 (1.34)	0.035	7.43 (1.22)	8.00 (1.24)	0.241
Innovates	6.26 (2.01)	7.89 (1.34)	0.001	7.58 (1.44)	8.67 (1.37)	0.03	7.00 (1.30)	7.93 (1.44)	0.013
Establishes Stretch Goals	5.63 (2.11)	7.78 (1.40)	<0.001	7.33 (1.78)	8.42 (1.68)	0.084	6.79 (1.25)	8.14 (1.23)	0.002
Communicates Powerfully and Prolifically	7.30 (1.35)	8.30 (1.27)	0.005	8.25 (1.66)	8.67 (1.67)	0.392	7.14 (1.29)	8.29 (1.33)	0.029
Gives permission for others to challenge	6.93 (1.69)	8.41 (1.37)	0.002	7.58 (1.98)	8.67 (1.50)	0.065	6.29 (1.44)	8.14 (1.29)	0.001
Transfers learning, understanding and value to colleagues	7.19 (1.52)	8.44 (1.22)	0.001	8.08 (1.38)	8.58 (1.51)	0.324	7.29 (1.33)	8.36 (1.15)	0.001
Provides evidence based & meaningful feedback	6.59 (1.87)	7.93 (1.24)	0.001	7.83 (1.90)	8.83 (1.34)	0.06	6.79 (1.31)	7.86 (1.51)	0.046
Acknowledges input of others to leadership learning	7.96 (1.16)	8.89 (1.12)	0.012	8.25 (1.77)	8.92 (1.51)	0.087	7.50 (1.61)	8.50 (1.02)	0.033

^†^ Missing data were excluded pairwise.

## Data Availability

The original contributions presented in this study are included in the article. Further inquiries can be directed to the corresponding author(s).

## Appendix B. L-EPP Learning Objectives

1. To assist participants with professional development, clinical progression and professional growth and goal achievement
2. To assist participants in developing lifelong learning capability in the area of leadership
3. To engage participants in active learning as an approach for focusing professional effectiveness in the practice setting
4. To identify areas of need and address them using a structured and outcome-related educational framework to improve patient and staff safety
5. To improve organisational leadership capacity within the nursing and midwifery workforce
6. To recognise and utilise leadership capabilities skills and knowledge in clinical practice, leading others in practice
7. To develop knowledge and skills in using leadership facilitation frameworks to gain self and situational awareness to assist in practicing in a leadership role
8. To identify participants’ own strengths and develop their knowledge and skills in critical self-reflection, to inform areas for own self-development relating to self and emotional awareness
9. To reflect on participants’ own learning in an environment of high support and high challenge
10. To identify ways of working to support group and team collaboration
11. To develop communication strategies for engaging with teams in collaborative ways of working
12. To understand the benefits of a multi-disciplinary team in leadership
13. To demonstrate increasing positive leadership qualities in practice
